# Development of Healthcare Service Design Concepts for NICU Parental Education

**DOI:** 10.3390/children8090795

**Published:** 2021-09-10

**Authors:** Hanui Yu, Dahae Woo, Hyo Jin Kim, Minyoung Choi, Dong Hee Kim

**Affiliations:** 1 Department of Industrial Design, Sungshin University, Seoul 02844, Korea; nuiii@daum.net (H.Y.); dahaewoo@naver.com (D.W.); 2 Graduate School, Seoul National University, Seoul 03080, Korea; hj.kim20916@gmail.com; 3 Department of Service Design Engineering, Sungshin University, Seoul 02844, Korea; minychoi@sungshin.ac.kr; 4 College of Nursing, Sungshin University, Seoul 01133, Korea

**Keywords:** education, healthcare service design, neonatal intensive care unit

## Abstract

The objective of this study was to develop healthcare service design concepts through an empirical study utilizing design thinking to improve the quality of caregiver education provided in the neonatal intensive care unit (NICU). This study adopted the Double Diamond Process of service design comprising the discover, define, and development stages. We identified 7 issues, organized into 10 healthcare service design concepts associated with NICU education: improving the design of educational material, improving materials for high-risk infant guidance, a practicum kit, a parent proficiency checklist, a systematic parent education manual, predictable guidelines for tests and treatment plans, waiting time that provides comfort, message cards that convey feelings, a reservation system for visits, and a post-discharge information sharing platform. The service concepts’ effectiveness was verified through evaluations by healthcare experts. The results represent customers’ perspectives and experiences regarding parental education. The application of the healthcare service design method could be further developed in future studies. The 10 service concepts derived from this study can be applied and evaluated as specific NICU educational programs.

## 1. Introduction

Parents of babies admitted to the neonatal intensive care unit (NICU) experience a significant sense of loss and helplessness that they were unable to give birth to a healthy baby [1].

Parents of babies in the NICU have a higher demand for information and guidance compared to those of typical mature infants as they have concerns regarding the vulnerabilities and potential disabilities of high-risk infants and, thus, are less likely to perceive situations positively [1,2]. Appropriate educational interventions can reduce parenting stress and raise childrearing confidence; therefore, systematic ongoing education is always being emphasized [3]. NICU education provided in hospitals includes information on the NICU environment and the concept and characteristics of high-risk infants in the section relating to hospitalization; in the discharge education section, information is provided about caring for high-risk infants, management of typical and special conditions, development of high-risk infants, and emotional support for parents [4,5]. The groups developing or providing educational programs were varied, including physicians, nurses, physical therapists, and occupational therapists, and they sometimes worked as teams [6,7].

However, parents’ viewpoints as customers have not yet been carefully considered [7,8]. This underscores the need for customized educational programs that reflect the needs of parents participating in the care of high-risk infants and maximize the educational impact through information accessibility, parental preferences, and diversity of educational media [7].

Patient education in healthcare service is effective when its process is patient-centric [9,10]. Service design, which differs from previous healthcare systems, is considered an effective method for understanding patients’ values and experiences [11,12]. Healthcare service design is the method of identifying the potential needs of various stakeholders using contextual research methods for all elements and paths experienced by health industry stakeholders through healthcare services and developing them through creative and cooperative design methods [13].

Healthcare service design can be divided into physical and non-physical elements. Physical elements refer to the environment and can be divided into the external environment, such as the exterior of hospital facilities, signage, and surrounding environment, and the internal environment, such as the interior design of hospital wards and waiting rooms, lighting, and uniforms of personnel. Non-physical elements include the act of preparing various channels, such as designing new experiences, multimedia, and guidance materials [14].

To date, healthcare service design implementations have tended to focus on local design improvements, such as spatial improvements for comfort, improvement of the treatment environment for comfort and sensitivity, improvement of service flow to shorten waiting time, and improvement of signage to prevent safety incidents [15]. Therefore, we adopted a comprehensive healthcare service design by including physical and non-physical elements and focused on parental education in the NICU. This study aimed to develop healthcare service design concepts to improve the quality of parental education in the NICU.

## 2. Methods

**Figure 1:**
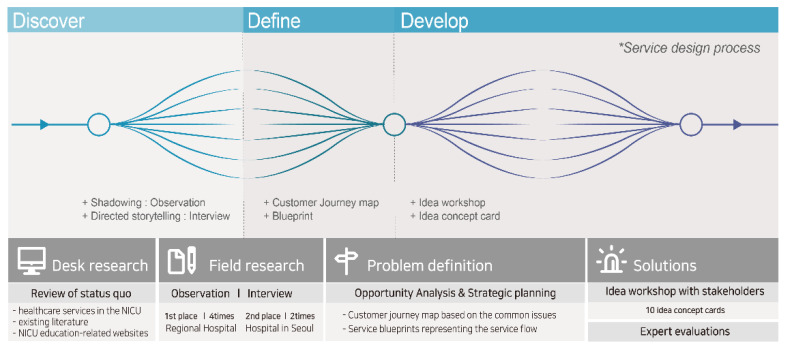
Research methods based on service design of this study.

Based on the Double Diamond Process [16], we utilized the Discover phase for defining, researching, and understanding the target situation; the Define phase for defining the problem to be solved; and the Development phase for solution development (Figure 1).

In the Discover phase, we reviewed the status of NICUs using observation methods such as the fly-on-the-wall method and shadowing and conducted general interviews and direct storytelling in two NICUs—one located in Seoul and the second in Suncheon. In the Define phase, customer journey maps and service blueprints were developed according to the service flow, identified from the data organized in the previous phase. Touchpoints identified in this process were used to group and identify opportunity elements and establish design strategies. In the Development phase, we held a workshop in which stakeholders and customers participated and engaged in brainstorming for idea proliferation. We analyzed the contents derived from this process and developed a healthcare service design concept, using the idea–concept cards method. The service concepts developed in this phase were verified for effectiveness through evaluations from design and healthcare experts.

Written informed consent was obtained from all participants as an inclusion criterion. The study protocol was approved by the Severance Hospital Institutional Review Board (4-2019-0078).

### 2.1. Discovery

#### 2.1.1. Review of Status Quo (Existing Data)

To identify the current state of healthcare services in the NICU, we researched existing literature and NICU-education-related websites.

The literature review was conducted between April and July 2019. The research period was set to 10 years (2010–2020) to focus on recent data. We searched using the search terms “parent education,” “neonatal intensive care unit,” “preterm infant,” “NICU education,” “high-risk infant education,” and “low-birthweight infant education” on PubMed, CINAHL databases, the Korea Education and Research Information Service, and the National Assembly Library. We selected 13 papers that were written in English or Korean and were suitable for the purposes of this research.

Furthermore, we conducted research through a variety of paths to identify the various experiences of actual parents, including not only academic papers but also personal blogs, online communities relating to high-risk infants and childrearing, news articles, Statistics Korea, and the Health Insurance Review and Assessment Service.

#### 2.1.2. Field Research

To understand the behavior of the parents’ healthcare services usage, we selected one NICU in a regional hospital and one located in a hospital in Seoul using convenience sampling for field research. We ensured that the research results reflected the differences in the geographic and ethnographic characteristics in regional and metropolitan areas, size of hospitals, and differences in the severities of patients. The main objective of field research was to derive touchpoints about the users’ experiences of healthcare services and identify pain points from the perspectives of parents and then to utilize them as opportunity elements for design. Furthermore, we aimed to examine all education-related services used in the NICU, including simple information transfers.

#### 2.1.3. Observation

Observations were conducted in the 30 min visiting time slots scheduled twice per day, which were the only touchpoints between the parents, babies, and the healthcare provider. Four observations took place in the regional hospital in April 2019, and two observations took place in August 2019 in the hospital located in Seoul. Depending on the situation, the observation methods were either fly-on-the-wall observation or shadowing. The observations focused on the contextual situations, conversations, and emotional components in terms of the interactions and events occurring inside and outside the NICU. The observation frames were time, place, related persons, content, atmosphere, and issues; specific observation points were behaviors of parents and healthcare providers, situational analyses, and educational methods.

The observations were recorded in notes. Through meetings, two of the researchers organized the behaviors of the parents based on space and time, identified contextual situations according to behavior, and categorized stakeholders who were directly or indirectly involved with the touchpoints.

#### 2.1.4. Interviews

Interviews were conducted to examine the situation of the healthcare providers and the parents based on our recorded observations. These interviews took place in meeting rooms and rest areas to avoid interrruption, each lasting between 30 min to an hour, with four parents, two doctors, and four nurses. First, information about the healthcare service system was collected from the healthcare providers; we aimed to understand their situation through their experience of cases involving infants and parents. We conducted interviews for parents of infants who were hospitalized or undergoing outpatient care after discharge, aiming to understand the emotional and psychological factors relating to hospital use, the information provided, their satisfaction associated with the education, and whether they needed any further information. In particular, the parents of infants who were already discharged were questioned regarding any issues that might have been experienced after discharge, namely whether they faced any difficulties due to information that they had not received or what additional information should have been included. We used the directed storytelling method for interviews relying on the thoughts and experiences of patients. The interviews were analyzed using an analysis matrix, and we organized issues based on common parental responses.

### 2.2. Definition

#### Opportunity Analysis and Strategy Planning

A customer journey map was created based on the common issues found in the observations and interviews. Customer journey maps are designed to visualize unseen experiences to identify complexities and problems. The parents under observation were grouped into similar cases and were given representative personas. Service flows (the process of using the services) for each persona were recorded on a timeline. We identified problems by recording the emotional changes experienced by the personas throughout the service flow and direct/indirect contact with stakeholders—including physicians, nurses, administration staff, nursing assistants, and cleaning staff—and connected their direct/indirect interactions with lines to identify pain points that can occur in the process as well as touchpoints for problem solving.

Furthermore, the service blueprints of the service flow were prepared by two experts in service design and two experts in nursing, independent of the customer cases. The service blueprint [17] is a chart that lays out the services according to the flow of experience from the customer’s perspective and lists interactions with various stakeholders who provide those services. It is composed of activity elements including the main actor performing the action, the party on the other side of the action, third parties and others relevant to the action, the target of the action, the tools used in the action, the action itself described with an action verb, and action-related situations. Through the service blueprint, we identified the touchpoints between the consumer and the provider and derived design issues to develop service concepts. We then defined the issues associated with the service around the derived touchpoints and issues, grouped the issues using the KJ Technique, identified opportunities, and established the design strategies [18].

### 2.3. Development

#### 2.3.1. Ideation

An ideation workshop was conducted to expand on various ideas. Using brainstorming and card sorting methods, two service designers, three NICU nurses, and two parents identified issues and points for improvement of educational services based on their experiences. The workshop involved the creation of a work board where situations, problems, and solutions could be recorded according to the healthcare service processes in the NICU. The participants were able to freely write their opinions on memo pads to be attached to the work board. The contents were classified and grouped into similar and overlapping opinions, and opinions with the same objective were grouped using the card sorting technique. The participants were asked to place stickers on the problems that required improvement as well as the most urgent and important solutions. By principle, participants were able to assign one vote to one opinion, but the total number of votes was not limited. This process was conducted to examine a variety of opinions that were considered important, confirmed by having the most votes.

#### 2.3.2. Service Concept

The ideas derived from the idea workshop were reprocessed to match the opportunity elements, and design strategies and were used in developing the healthcare service design concept by using the idea–concept cards method. A design idea–concept card is a card with design ideas and related content created in accordance with the service concept to be developed. As the cards contain subjects associated with ideas and contextual information about how the ideas would be developed, they can be shared with multiple designers and are structured to facilitate idea descriptions for the development of specific designs [15].

Expert evaluations were conducted from 15 January to 17 January 2020 to verify the validity of service concepts and assess their respective importance. A total of five experts participated, including two nurses, one nursing professor, one doctorate-level researcher, and one professor of service design. They were invited to evaluate the 10 service concepts on a seven-point scale using a 15-question survey. The evaluation items included eight questions on the evaluation elements of service values and consumer satisfaction, which were harmony, differentiation, effectiveness, reliability, satisfaction, and importance and seven questions on the evaluation elements of service execution and maintenance, which were economics, sustainability, and feasibility. The questionnaires and relevant material for evaluation were distributed to and returned by participants. The average scores for each question were calculated.

## 3. Results

Based on the results of the observations and interviews, we ascertained the key issues of each group regarding services and parents’ needs (Table 1).

### 3.1. Definition

**Figure 2:**
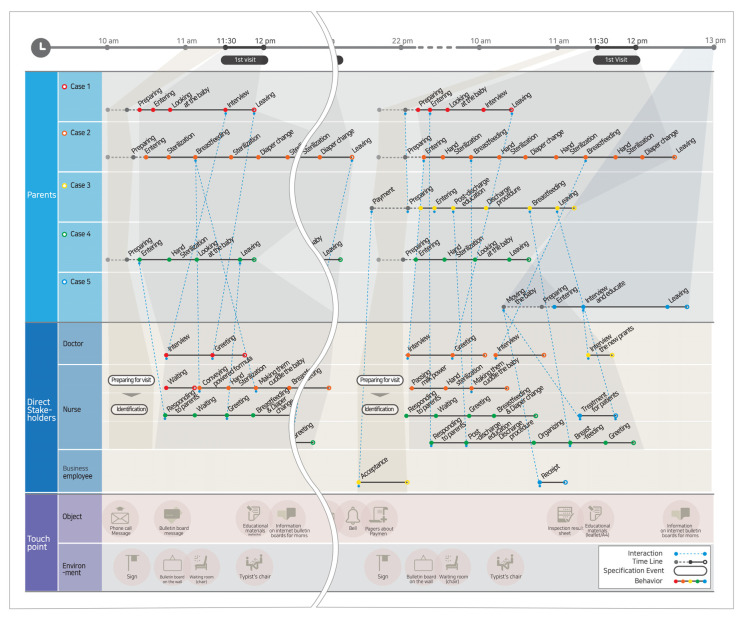
Customer journey map: A map showing the entire process of experiencing the NICU’s services so that parents can understand who they are meeting, what they are doing, and what interactions and contact points they should expect.

**Figure 3:**
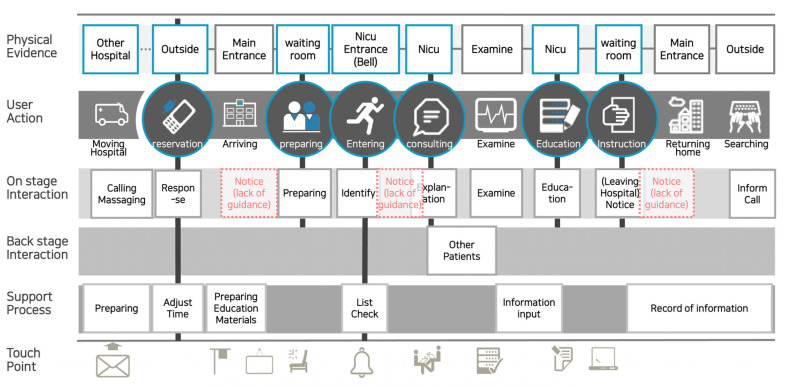
Service blueprint: A layout of services demonstrating the flow of experience from the parents’ perspective that outlines interactions with various stakeholders who provide the services.

By visualizing services through a customer journey map and service blueprint, we were able to confirm touchpoints in accordance with the overall service flow, such as the emotional factor of anxiety and discomfort that can occur in the NICU, the physical environment, on-stage interaction, backstage interaction, support processes, and user actions (Figure 2 and Figure 3).

In this process, based on the design strategy, we presented the following four directions for service concept development to improve parental education: First, enhancing the educational material with experience-based content. This entailed improving the contents of the educational material, depending on its purpose and objective and fundamentally improving the quality of education; predicting the educational content needed depending on the parents’ experiences and situations; and, in case education could not be provided, substituting similar educational material for practice. Second, developing educational materials sensitive to the parents’ needs, which involved reorganizing data and using manuals to maximize the positive effect of education; proposing appropriate delivery media and methodology depending on the educational content and organizing the design to be visually effective; in addition, possibly utilizing a checklist for education or manuals. Third, encouraging psychological communication with the healthcare providers requiring training in meeting emotional needs such as offering comfort and verbal reassurance to parents using quotes, for their psychological stability; presenting touchpoints to the healthcare provider to ensure that parents better understand their communications; and providing parents with full support to increase their trust in treatment. Fourth, establishing an efficient operating system that entails increasing work efficiency and reducing unnecessary waste. This required improvements in processes, such as applications for appointments and testing in other departments, as well as improving system efficiency, such as supplementing the nurse shift systems. These four directions for service concept development were used as strategies to determine the healthcare service design concepts.

### 3.2. Development

We identified seven types of issues in NICU parental education through the ideation workshop based on the results of the previous process (Table 2).

The final phase was to utilize the four idea concepts and seven issue types to develop the design strategy. This involved developing improved educational materials and a parent education manual, psychological consideration for the parents, and improving operational systems to increase work efficiency. Using the idea–concept cards, a total of 10 healthcare service design concepts were developed (Table 3). The idea–concept cards included an existing problem, a solution that could lead to improvement by using the idea, and the idea’s limitations.

### 3.3. Validation of Design Strategy and Healthcare Service Design Concepts

The expert validity analysis yielded a score of 5.6 out of 7, with an overall positive average rating. The service concepts associated with relatively low requirements of cost and time, such as visual improvements to materials and supplementing educational materials, had higher scores than service concepts associated with high workload and expenditures, such as excessive workload for nurses, improvements to facilities and environments, and system developments (Table 4).

## 4. Discussion

NICU services are focused on professional and urgent medical needs for premature infants. Therefore, efficiency and effectiveness of the activities of healthcare providers are considered extremely important, often resulting in customer-centered care being dropped from the list of priorities.

One of the important discoveries, based on the Double Diamond Process in this study, was that the existing approach still had touchpoints that, if addressed, could mitigate parental challenges such as stress, anxiety, discomfort, and wanting accurate information. The study results were based on a customer-centered viewpoint, and the education service concept was derived from the parents’ emotional touchpoints and the delivery of accurate and effective information.

Second, we identified several issues on which the parents of babies in the NICU must be supported and trained. As for the main contents of education, parenting skills that require practice (such as bathing and breastfeeding) and health management of babies (such as emergency response and medication) have been presented as shown in previous studies [19,20,21]. In addition to identifying the contents, we suggested methods for parents to understand and familiarize themselves with these contents by considering the design aspects of educational materials, using a practical training kit, and developing a checklist; this can be used as a more effective parental education strategy. Providing guidelines for testing and treatment plans and establishing a reservation system for visits can play a role in reducing uncertainty and anxiety in parents as well as creating systematic education [22,23]. Providing message cards or offering comfortable waiting time can maximize education while also considering parents’ emotions. Support systems are known to be an important resource for parents [24,25]; therefore, we suggested a way to easily access informational and professional support. This emphasizes the importance of continuing education after discharge, and it will be an important educational strategy for parents to practically solve various problems that may arise while raising their children

Third, we found that, for the physically and mentally exhausted parents, all the experiences in the NICU were opportunities for education, including meetings with healthcare providers, the overall environment, and formal education sessions. Thus, healthcare providers need to consider every parental visit as an opportunity and time for education. High-quality services besides medical activities could be provided by always improving touchpoints everywhere.

Fourth, this study aimed to provide healthcare service experiences that satisfy the parents’ needs and medical objectives, using a customer-centric service design based on design thinking. We used observations and interviews from healthcare providers’ work to suggest possible educational methods that they can adopt. Our results suggest that the needs of both sides need to be considered.

Finally, this study effectively used the service design method for the development of a customer-centered educational intervention. Service process innovations that find and respond to the touchpoints of difficulties that customers usually experience can be promoted by analyzing “service design methodology,” based on the experience of patients. We also applied the service design method to conduct an empirical study, from reviewing the status to field research that investigated the overall range of healthcare services in the critical and closed space of a NICU. We identified realistic questions and solutions through workshops, in which parents participated positively, to emphasize intervention-based parent experience. In addition, this study did not limit the development of healthcare service design to physical and non-physical elements; rather, it focused on service designs that provided guidance for parents of hospitalized infants and education for the post-discharge period, helping to comprehensively plan for subject-focused services.

Through this process, we identified 10 parent-centric healthcare service design concepts for more effective delivery of education. The results of this study can contribute to strategies emphasizing the perspectives and experiences of the consumers of parental education, which had originally focused on the roles of professional healthcare providers as the providers of education [26]. This extends the existing limited educational content and strategies [7]. In addition, the service design concepts presented the existing problems and limitations together with the corresponding solutions, so that, when applied to clinical practice, they could be modified and implemented to suit each on-site situation.

Furthermore, this study is significant as the results are driven by flexible design thinking that has received positive feedback from expert evaluation, including experts in clinical practice and education. However, our study also has some limitations. The developed design concept cannot be applied to actual parent education. Owing to the lack of actual parental education, repetitive issues and concepts in this study could not be summarized, and parental satisfaction could not be measured. Therefore, we recommend that the service concepts, which have been positively evaluated in accordance with their core values through a detailed analysis, should be developed, applied, and evaluated with specific educational programs, including parents’ satisfaction evaluation in a future study.

## 5. Conclusions

The results of this study suggest the value of developing comprehensive patient-centered education for parents with babies admitted to the NICU, using the service design method. Our focus was on understanding the healthcare service experiences of parents, analyzing the services from a contextual viewpoint, and recommending healthcare service design concepts that can meet the needs of the parents. This is a significant attempt to provide high-level services that consider the parents’ needs, going beyond services for routine treatment.

We obtained 10 service concepts from this study comprising problems, solutions, and limitations. When applying these service concepts to a clinical setting, the problems, solutions, and limitations should be considered and modified accordingly. We suggested comprehensive service concepts that include physical and non-physical elements. These could be applied to new and varied interventions in clinical settings and may be used as effective intervention strategies, as they contain educational content and methods that value patient experience, which is not the case with the existing provider-centered education methods.

## Figures and Tables

**Table 1:** Issues of key stakeholders and needs of parents.

Customer Issues		
Parents	Situation	Increased fatigue and anxiety of the mother. Always feeling vulnerable, despite being the customer. Difficulties in understanding professional medical terms and in identifying the baby’s conditions.
	Interpretation	Increased anxiety and sensitivity in the postnatal period. Being sensitive to the nurses because the baby is in the nurse’s care. Collecting information from the internet and specialty literature to address their unresolved questions.
Service Provider Characteristics		
Doctors	Situation	Clear and objective explanations are required. Parents’ interviews are conducted only on request. Necessary to persuade parents to allow their babies to receive treatment.
	Interpretation	Realistic explanations are more important than vague expectations. Strong support from the parents but lack of opportunities to meet with specialists. Important to build trust.
Dedicated education nurse	Situation	Having numerous private questions after the discharge of the baby. Handover of information related to sensitive parents.
	Interpretation	Repetition of basic questions from parents. Requires a manual to assist parents.
General nurse	Situation	Parental education takes place for a limited period of time. Frequent nurse rotation due to shift work. Difficulties facing fatigued parents.
	Interpretation	All education must be completed within the visiting hours. Poor educational efficiency due to frequent nurse changes. Emotional difficulty in dealing with parents.

**Table 2:** Seven issue types in NICU parental education.

Issues
1. Lack of understanding and consideration for parents 2. Physical limitations, including facilities and environment 3. Operating systems that hinder work efficiency 4. Psychological burdens in a desolate atmosphere 5. Lack of systematic protocols for education 6. Lack of medical information7. Confusion regarding unforeseen situations

**Table 3:** Healthcare service design concept (idea–concept cards).

1. Improving the Design of Educational Materials	
Unified design (size, format) for ease of understanding and storage. Provide visualized data for content that requires visual understanding (text, image, or video). Provide workbooks for content that requires exact measurement such as breastfeeding and medication management.	
Problem	Difficult to manage educational materials as they have different sizes and formats. Effective teaching methods differ according to the content of the education.
Tangible action	Increase the level of understanding by unifying the design. Use effective information delivery methods for different educational content (text/image/video). Important and frequently viewed content to be provided as posters, rather than booklets, to be posted on walls.
Limitations	None
2. Improving Guidance Materials for the Care of High-Risk Infants	
Provide guidelines on caring for high-risk infants after discharge, depending on corrected age. Provide self-check material so that the parents can assess the situation themselves. Provide guidance on changes and accompanying methods of change throughout growth and development, such as changes to general milk powder and breastfeeding amounts. Provide Q&A for emergency and manageable situations.	
Problem	High volume of everyday guidance due to changes in growth and development. Difficult to check the specifics of growth and development of the baby. There are not as many emergencies as inquiries relating to abnormal symptoms displayed by babies.
Tangible action	In addition to examinations of infants and toddlers, distribute material on growth and development, allowing for constant self-checking. Provide a recording platform on the website, to allow for its use as reference data during outpatient visits.
Limitation	Using websites to record inquiries requires an electronic records management system linked with medical treatment.
3. Practicum Kit	
Educational kits to be placed inside and outside the NICU, which may be used while waiting during visiting hours. Parents will use them directly and ask questions, allowing them to obtain practical knowledge. Kit contents are systematically constructed to ensure easy content acquisition and utilization.	
Problem	As education is provided verbally as a one-time event, on paper and via video, it is difficult to accurately understand it and put it into practice. Queries occur in the process of childrearing after discharge.
Tangible action	Place items used in childrearing out during visiting hours so that the parents are free to utilize them. Practice activities, such as medicine dosage. The overall image of the education can be conveyed using the items inside the kit.
Limitation	Sufficient use of the kit is impossible as visiting hours are short. Costs associated with developing and making the kits available.
4. Parent Proficiency Checklist	
Create a list of contents for education, enter the names of educators and parents to establish accountability. Allow for the baby’s discharge after the parents are fully proficient in the educational content, move to the next stage, or repeat the education depending on the proficiency of the parents. Checklist to be provided to both the hospital and the parents; special education to be mandatory for both parents.	
Problem	Depending on the difference in the skills of education providers and consumers, discharges may happen without the educational content having been fully understood. Parents are unaware of the education they have, or have not, received. Parents are not aware of the type of education they require. Parents have received CPR education but feel that they would not be able to do it in emergencies.
Tangible action	Create a mandatory education list for each parent and check off its points as education is provided. Confirm the level of understanding with parents, repeating education when necessary. Adjust the discharge schedule depending on the level of education absorbed.
Limitation	Visiting hours are limited and multiple parents must be educated. Additional personnel may be required to systematically manage and deliver education. Given individual differences in acquisition of knowledge and poor participation, discharges may be delayed.
5. Systematic Parental Education Manual	
Organize the education manual for effective parental education. Include parental communication and response guides on the content of the education, number of educational programs provided, targets of mandatory education, evaluation of learning ability, communication methods, and speaking styles. Make notes on customized information (characteristics, tendencies, and habits of the baby) and guide the parents through these during education, to increase credibility.	
Problem	Each healthcare provider renders different information during visits, leading to confusion. Nurses change often, leading to repeated or omitted information. Some parents are hurt by the tone of voice used by healthcare providers. Nurses have difficulties in dealing with parents.
Tangible action	Unify basic education and coping manuals for parents’ questions and use them when communicating with parents. Through the education manual, parents can obtain consistent information, and nurses can appropriately respond to difficult situations with parents.
Limitation	Heavier workload for nurses as they must understand the manual. The manuals cannot be used to respond to unexpected situations.
6. Predictable Guidelines for Testing and Treatment Plans	
Provide visualized overall plans on testing and treatment for the parents. Include information, side effects, and results of treatments and tests accordingly.	
Problem	Parents are always anxious as they are not able to predict the situations that may arise in the coming steps. Lack of information on the treatment and testing underway makes searches difficult as parents do not know what they are searching for.
Tangible action	Common testing and treatment plans to be provided for typical cases of high-risk infants, allowing the parents to be aware of and prepare for important information.
Limitation	Difficult to apply specific cases for each baby. Risk of increasing confusion for parents if the situation differs from the plan.
7. Waiting Time that Provides Comfort	
Use affordance design to allow the parents—even those visiting for the first time—to adapt to the situation easily. Place comforting phrases in different places to create a warm environment, helping the parents to feel comforted. Use a text-based, interactive medium (for example, a question board) so that the parents are free to ask questions and receive answers from the healthcare provider as they wait. Adjust visiting hours if delivery rooms and the NICU are attached to one another in the hospital.	
Problem	Parents of high-risk infants are likely to experience negative emotions, such as anxiety, guilt, despair, and depression. The waiting area is small and the movements of parents are limited. Feeling uncomfortable as other people stare at them while they wait. Notices filled with medical terms create a fearful atmosphere.
Tangible action	Organize flow of movement through affordance design. Adjust visiting hours if delivery rooms and the NICU are attached to one another, reducing emotional burden for the parents. Use warm and sympathetic phrases to comfort and cheer fatigued parents. Create a question board to provide a participatory platform where parents can ask questions and receive answers. Parents can share common questions and answers, resolving the issue of unnecessary repetitions.
Limitation	Expenditure associated with creating a comfortable environment. May be difficult to implement given the spatial conditions in the hospital.
8. Message Cards that Convey Feelings	
Give cards to parents so that they can write to the healthcare provider and communicate with them freely. Attach general information on topics such as baby’s body weight and feeding records to the bed so that the parents can freely check it during visits.	
Problem	Parents have trouble communicating as they feel the healthcare provider is too busy. Wanting to check daily records of the baby, such as weight, temperature, and feeding volume, even if they do not know much about the information.
Tangible action	Message cards can act as touchpoints with the healthcare providers who are hard to meet with, such as head nurses or specialists. The parents can be comforted in the process of honestly laying out words that can be difficult to convey directly, which can reduce unnecessary conversations and waste of emotions.
Limitation	Possible increase in the burden on healthcare providers due to the message cards.
9. Reservation System for Visits	
Develop a simple visit scheduling system in consideration of the positions of both nurses and parents.	
Problem	Depending on the situation, it is possible that parents may misunderstand the nurses asking about their visits. Nurses need to be aware of the number of visitors to prepare for their work allocation and responses to parents. Parents need to go through nurses to apply for appointments.
Tangible action	Easier application process for appointments through a simple online system, rather than phone calls that can be misleading. Nurses can determine the number of visitors, and parents can check for the number of visitors and change visiting hours. Parents can apply for appointments with specialists in a simple manner.
Limitation	Issues associated with development and maintenance costs.
10. Platform for Post-Discharge Information Sharing	
Create a Q&A section that parents can use to easily ask questions after discharge. Create a platform for parents of discharged babies, allowing them to exchange information with each other. Provide emergency contact guides.	
Problem	Increased workload for nurses due to big and small inquiries from parents. After discharge, parents face real-life childrearing challenges and develop numerous questions. They access experience-based, incorrect information from the internet and respond based on such information.
Tangible action	Post a Q&A section on frequently asked questions. Create an internal community to facilitate information exchange among parents. Expert responses promote reliability and a reduced workload for nurses.
Limitation	Difficulties in ongoing operations and management. Requires real-time responses. May face the risk of becoming a general online community.

**Table 4:** Expert validity.

Healthcare Service Design Concept	Mean	Overall Mean
1. Improving the design of educational materials.	5.88	5.60
2. Improving guidance materials for caring for high-risk infants.	5.81	
3. Practicum kit.	5.85	
4. Parent proficiency checklist.	5.92	
5. Systematic parental education manual.	5.57	
6. Predictable guidelines for testing and treatment plans.	5.69	
7. Waiting time that provides comfort.	5.52	
8. Message cards that convey feelings.	5.33	
9. Reservation system for visits.	5.21	
10. Post-discharge information sharing platform.	5.16	
